# SAHA Decreases HDAC 2 and 4 Levels *In Vivo* and Improves Molecular Phenotypes in the R6/2 Mouse Model of Huntington's Disease

**DOI:** 10.1371/journal.pone.0027746

**Published:** 2011-11-28

**Authors:** Michal Mielcarek, Caroline L. Benn, Sophie A. Franklin, Donna L. Smith, Ben Woodman, Paul A. Marks, Gillian P. Bates

**Affiliations:** 1 Department of Medical and Molecular Genetics, King's College London, London, United Kingdom; 2 Neusentis, Pfizer Inc., Cambridge, United Kingdom; 3 Cell Biology Program, Sloan-Kettering Institute for Cancer Research, Memorial Sloan-Kettering Cancer Center, New York, New York, United States of America; University Medical Center Groningen, Netherlands

## Abstract

Huntington's disease (HD) is a progressive neurological disorder for which there are no disease-modifying treatments. Transcriptional dysregulation is a major molecular feature of HD, which significantly contributes to disease progression. Therefore, the development of histone deacetylase (HDAC) inhibitors as therapeutics for HD has been energetically pursued. Suberoylanilide hydroxamic acid (SAHA) – a class I HDAC as well an HDAC6 inhibitor, improved motor impairment in the R6/2 mouse model of HD. Recently it has been found that SAHA can also promote the degradation of HDAC4 and possibly other class IIa HDACs at the protein level in various cancer cell lines. To elucidate whether SAHA is a potent modifier of HDAC protein levels *in vivo*, we performed two independent mouse trials. Both WT and R6/2 mice were chronically treated with SAHA and vehicle. We found that prolonged SAHA treatment causes the degradation of HDAC4 in cortex and brain stem, but not hippocampus, without affecting its transcript levels *in vivo*. Similarly, SAHA also decreased HDAC2 levels without modifying the expression of its mRNA. Consistent with our previous data, SAHA treatment diminishes *Hdac7* transcript levels in both wild type and R6/2 brains and unexpectedly was found to decrease *Hdac11* in R6/2 but not wild type. We investigated the effects of SAHA administration on well-characterised molecular readouts of disease progression. We found that SAHA reduces SDS-insoluble aggregate load in the cortex and brain stem but not in the hippocampus of the R6/2 brains, and that this was accompanied by restoration of *Bdnf* cortical transcript levels.

## Introduction

Huntington's disease (HD) is a progressive neurological disorder for which there is no effective disease-modifying treatment [1], [2]. The disease is caused by the expansion of a CAG repeat to more than 35 CAGs within exon 1 of the *HTT* gene. This leads to a wide-range of characteristic symptoms including personality changes, motor impairment and weight loss, which progress over the course of 15–20 years to death [3]. At the molecular level, mutant huntingtin has a strong propensity to self-aggregate and form a wide-range of oligomeric species as well as insoluble aggregates [4], [5], [6], [7] that lead to an imbalance in cellular homeostasis [8]. As a consequence, one of the major molecular features of HD is transcriptional dysregulation, which significantly contributes to disease progression [9],[10],[11].

Globally, transcription is regulated at the level of chromatin by a variety of epigenetic marks. This includes the covalent modification of conserved lysine residues within histone proteins and is orchestrated by histone acetylases (HATs) and histone deacetylases (HDACs). Mammalian HDACs are a family of 18 molecules divided into four groups based on structural and functional similarities: class I (HDACs: 1, 2, 3, 8), class IIa (HDACs: 4, 5, 7, 9), class IIb (HDACs: 6, 10), class III (sirtuins 1–7) and HDAC11 as the sole member of class IV [12]. In HD, it has been proposed that an imbalance in histone acetylation is caused by the inactivation of HATs [13],[14],[15]. Hence, abnormal histone acetylation and chromatin remodelling might be a key process leading to transcriptional dysregulation [16]. Therefore, much effort has been directed towards developing HDAC inhibitors as an HD therapeutic [17] and initial genetic studies performed in flies and worms have confirmed that these may have a significant potential [14],[18],[19].

Preclinical evaluation of the HDAC inhibitor suberoylanilide hydroxamic acid (SAHA) demonstrated a dramatic improvement of the motor impairment in the R6/2 mouse model of HD [20]. Initially, SAHA was shown to inhibit members of class I and class II HDACs at nanomolar concentrations [21], but is predominantly an inhibitor of class I HDACs as well as the class IIb enzyme HDAC6 [22],[23]. More recently, activity based probes have been used to demonstrate that SAHA can bind to both class I and IIa HDACs [24],[25]. Furthermore, it has been shown that in cancer cell lines, SAHA can lead to the degradation of class IIa HDACs 4 and 5 via RANBP2 mediated proteasome degradation *in vitro* [26]. We are currently exploring the potential mechanisms by which SAHA might act to improve HD-related symptoms in a mouse model of HD. In this study we have investigated whether the chronic treatments of SAHA might result in the degradation of class IIa HDACs *in vivo*.

## Results

**Figure 1:**
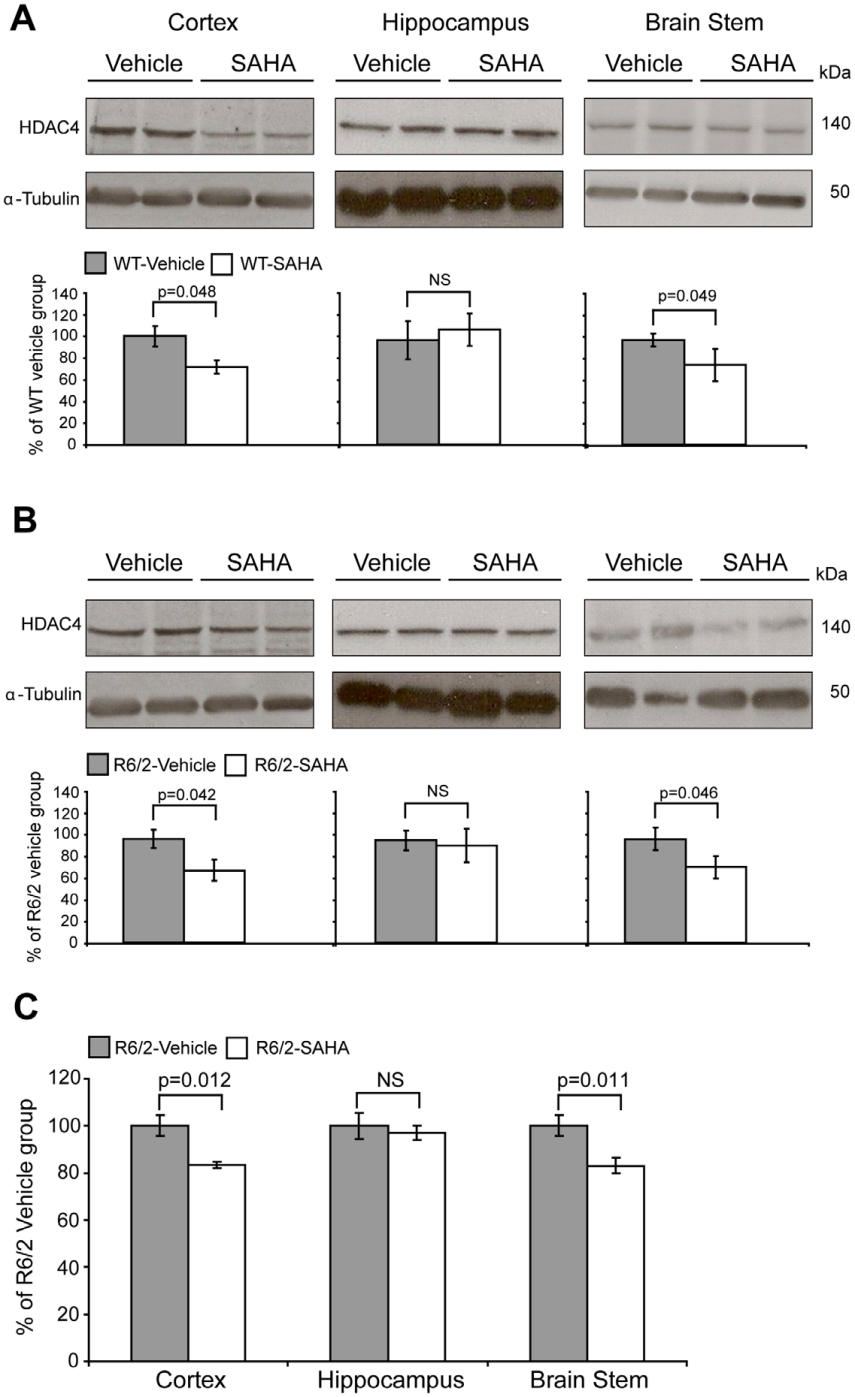
Chronic administration of SAHA decreases HDAC4 levels and reduces aggregate load – 1^st^ trial. HDAC4 protein levels are reduced in the cortex and brain stem, but not in the hippocampus of WT and R6/2 mice treated with SAHA. Representative western immunoblots of 20 µg of cortical, brain stem and hippocampal homogenates from 9-week-old WT (A) and R6/2 (B) mice treated with SAHA or vehicle, immunoprobed with the Sigma (Fig. 1A, 1B - brain stem) or the Santa Cruz (Fig. 1A, 1B – cortex and hippocampus) anti-HDAC4 antibodies are shown. HDAC4 protein levels were normalized to alpha-tubulin and quantified using densitometry. Representative graphs illustrate relative HDAC4 protein levels as a percentage of the respective vehicle group. Error bars represent S.E.M. (*n* = 4). The seprion-ligand ELISA was used to quantify aggregate loads, which were significantly reduced in the cortex and brain stem but not in the hippocampus of the R6/2 SAHA treated mice (C). Error bars are S.E.M. (WT vehicle: n = 6; WT SAHA: n = 6; R6/2 vehicle: n = 6; R6/2 SAHA n = 5).

In order to determine whether SAHA results in a reduction of class IIa HDACs at the protein level *in vivo*, WT and R6/2 mice were administered vehicle or SAHA in the drinking water (0.67 mg/ml) from five to nine weeks of age as previously described [20]. HDAC4 protein levels were measured in three different brain regions: cortex, hippocampus and brain stem (Fig. 1) by SDS-PAGE and immunoblotting with an N-terminal anti-HDAC4 antibody (Sigma) as well as with an HDAC4 antibody that had been raised against a peptide adjacent to the deacetylase domain (Santa Cruz). We observed a significant reduction in HDAC4 in the cortex and brain stem but not in the hippocampus of both WT (Fig. 1A) and R6/2 (Fig. 1B) mice treated with SAHA as compared to vehicle.

We have previously shown, that exposure to SAHA does not modulate the formation of huntingtin aggregates in organotypic hippocampal slice cultures prepared from R6/2 neonates [20]. At that time, we did not have the means to quantitatively determine the level of SDS-insoluble aggregates in brain tissue. However, since then, we have developed the seprion-ligand ELISA, a highly quantitative method with good statistical power that can be used to measure changes in aggregate load that occur *in vivo* in response to pharmacological or genetic manipulations [7]. Therefore we employed this assay to determine whether SAHA can modulate huntingtin aggregatation *in vivo*. We found that chronic treatment of R6/2 mice with SAHA significantly reduced aggregation in the cortex and brain stem but not in the hippocampus (Fig. 1C).

**Figure 2:**
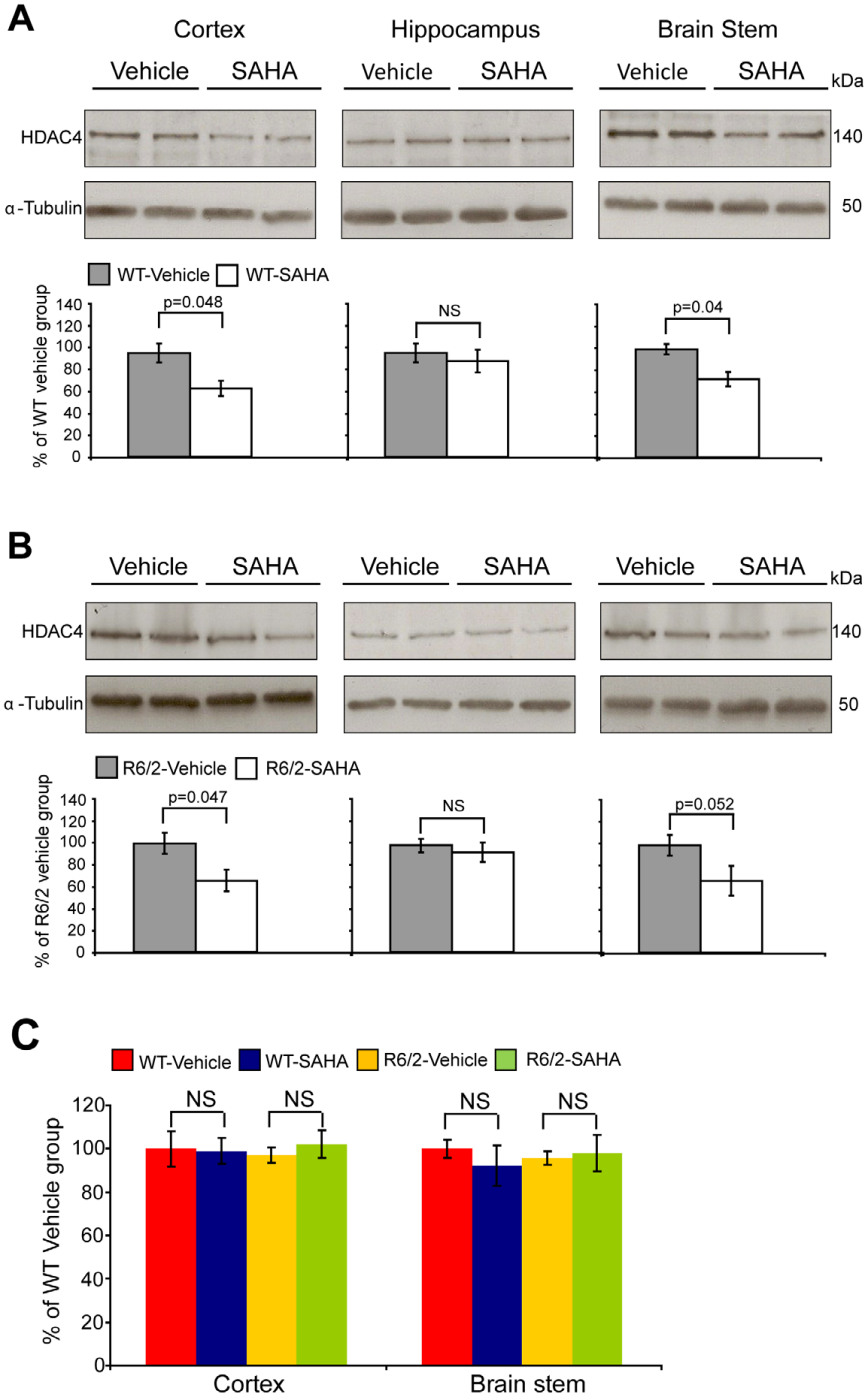
Chronic administration of SAHA decreases HDAC4 protein but not mRNA levels – 2^nd^ trial. HDAC4 protein levels are reduced in the cortex and brain stem, but not in the hippocampus of WT and R6/2 mice treated with SAHA. Representative western immunoblots of 20 µg of cortical, brain stem and hippocampal homogenates from 9-week-old WT (A) and R6/2 (B) mice treated with SAHA or vehicle, immunoprobed with the Santa Cruz anti-HDAC4 antibody are shown. HDAC4 protein levels were normalized to alpha-tubulin and quantified using densitometry. Representative graphs illustrate relative HDAC4 protein levels as a percentage of the respective vehicle group. Error bars represent S.E.M. (n = 4). C. *Hdac4* transcript levels were unaffected in the cortex and brain stem of the WT and R6/2 SAHA treated mice. *Hdac4* mRNA expression levels are shown as a relative expression ratio to the geometric mean of three housekeeping genes in the cortex and brain stem of 9-week-old WT vehicle (red bars), WT SAHA (blue bars), R6/2 vehicle (yellow bars) and R6/2 SAHA (green bars) mice. Error bars are S.E.M (n = 6). NS = not specific.

**Figure 3:**
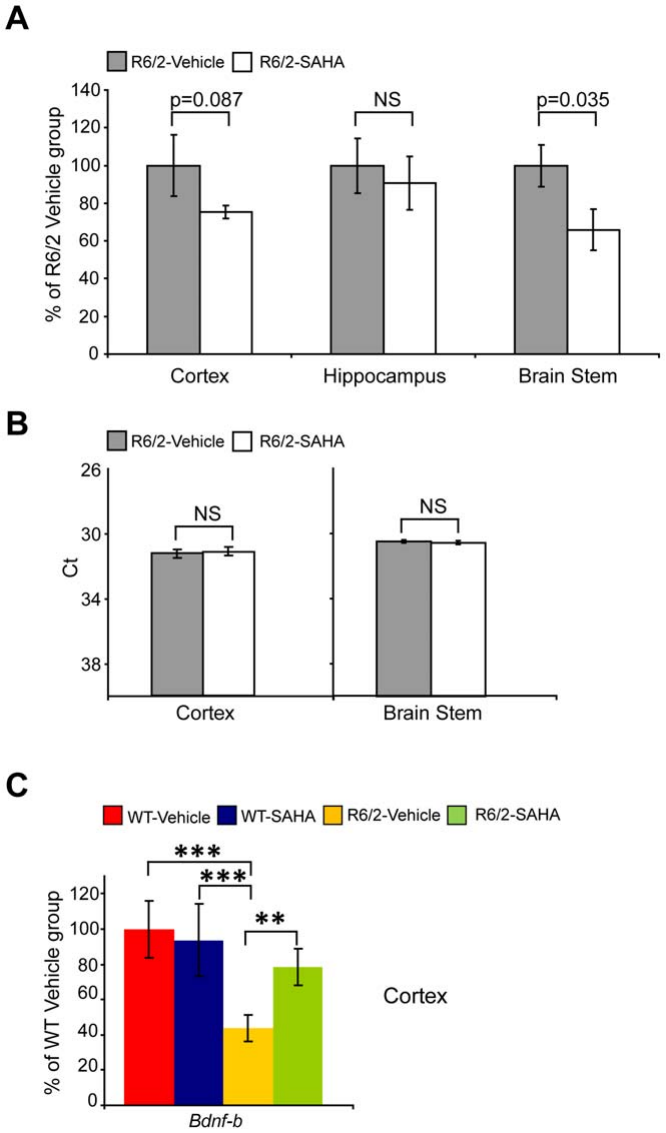
Chronic administration of SAHA reduces aggregate load and rescues *Bdnf* transcript levels without affecting mt-Exon 1 transcript levels – 2^nd^ trial. The seprion-ligand ELISA was used to quantify aggregate loads, which were found to be reduced in the brain stem and cortex but not in the hippocampus of the R6/2 SAHA treated mice (A). Error bars are S.E.M. (*n* = 6). Mt-Exon 1 transcript levels in the cortex and brain stem of R6/2 vehicle and SAHA treated mice were unchanged (B). Representative graphs show the crossing threshold (Ct) of the mt-Exon 1 transcript levels for R6/2 vehicle and SAHA treated mice. cDNA from vehicle and SAHA treated WT mice served as a negative control (data not shown). Error bars are S.E.M (n = 6). *Bdnf* transcript levels were partially rescued in the cortex of SAHA treated R6/2 mice but were unchanged in SAHA treated WT mice (C). *Bdnf* mRNA expression levels were assessed by qPCR and presented as a relative expression ratio to the geometric mean of three housekeeping genes in the cortex of 9-week-old WT vehicle (red bars), WT SAHA (blue bars), R6/2 vehicle (yellow bars) and R6/2 SAHA (green bars) mice. Error bars are S.E.M (n = 6). * p<0.05, ** p<0.01, *** p<0.001.

To confirm our results and extend our molecular analyses we performed a second trial that replicated the original dosing regimen. As found in the first trial, SAHA administration decreased HDAC4 protein levels in the cortex and brain stem but not in the hippocampus of both WT (Fig. 2A) and R6/2 (Fig. 2B) mice. We were intrigued to determine whether SAHA targets HDAC4 at the protein level or whether it modulates the *Hdac4* transcript. Due to the limited amount of hippocampal tissues, HDAC4 transcript levels were only assessed in the cortex and brain stem. Quantitative RT-PCR (qPCR) showed that there was no difference in *Hdac4* mRNA levels between vehicle treated WT and R6/2 mice and that SAHA did not affect *Hdac4* mRNA levels in either WT or R6/2 mice (Fig. 2C). The seprion-ligand ELISA confirmed that there was a significant reduction in SDS insoluble aggregate load in the brain stem of R6/2 treated mice at 9 weeks of age and a trend toward reduction in the cortex (Fig. 3A). Again, no change in aggregate load was found in the hippocampus (Fig. 3A). To ensure that the decrease in aggregate load had not occurred as a consequence of a reduction in the expression of the R6/2 mutant exon 1 huntingtin transgene (mt-exon 1), we performed qPCR and found that the level of the R6/2 trangene was not altered upon SAHA administration (Fig. 3B).

It is well established that brain derived neurotophic factor (BDNF) plays a crucial role in HD pathogenesis [27], that *Bdnf* transcript levels diminish during desease progression in the R6/2 mouse model [28] and that over-expession of BDNF ameliorates HD-related phenotypes in mice [29]. Therefore we were interested in determining whether SAHA might modulate *Bdnf* transcript levels. We used a qPCR assay designed to detect the levels of the BDNF protein coding region. As predicted, *Bdnf* transcript levels were reduced in the R6/2 vehicle treated group as compared to WT, and we found that its levels were partially restored upon SAHA treatment (Fig. 3C). Interestingly, SAHA did not affect *Bdnf* mRNA levels in WT mice as compared to those treated with vehicle.

**Figure 4:**
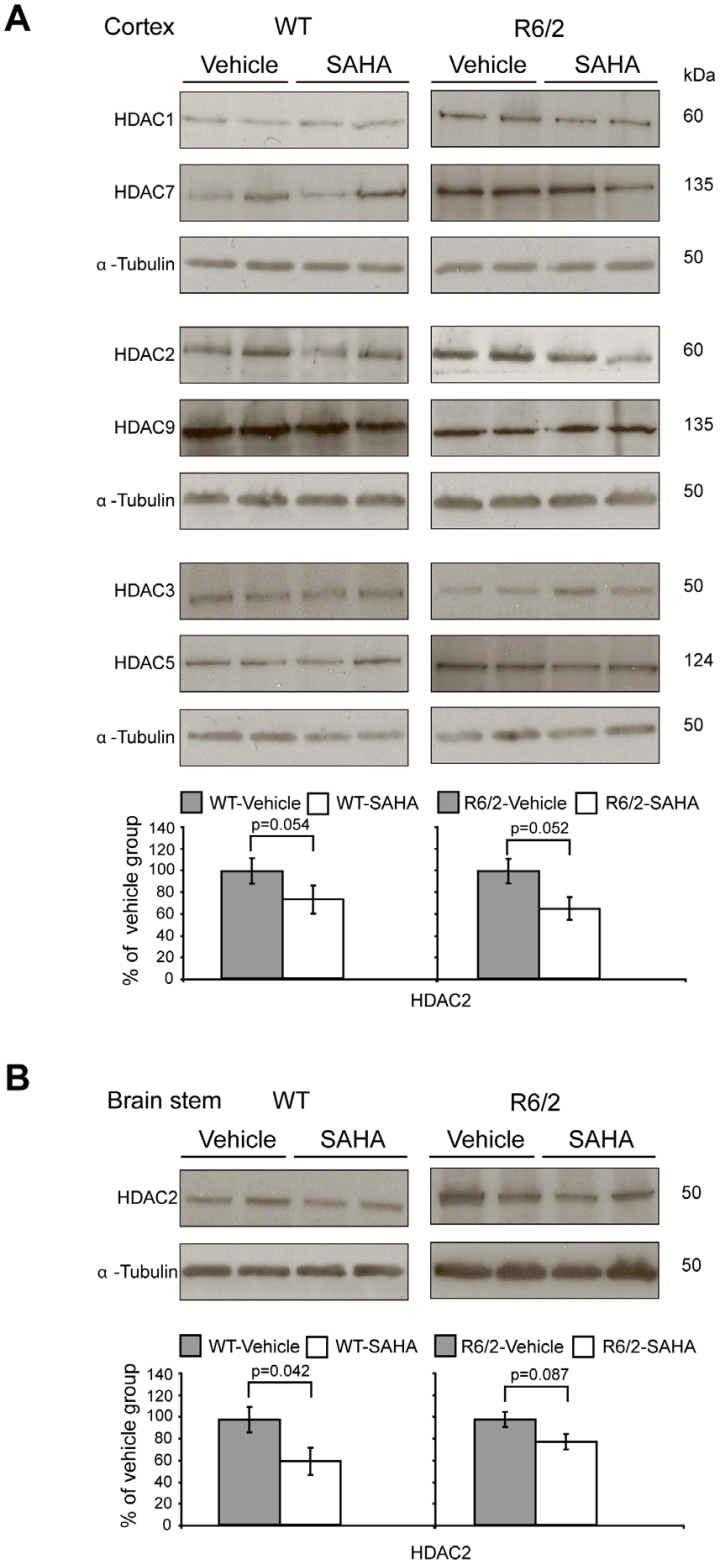
SAHA treatment decreases HDAC2 protein levels but does not affect the protein levels of other class I and IIa HDACs – 2^nd^ trial. HDAC2 protein levels are reduced in the cortex (A) and brain stem (B) but not in the hippocampus (data not shown) of WT and R6/2 SAHA as compared to vehicle treated mice. Representative western immunoblots of 20 µg of cortical and brain stem homogenates from 9-week-old WT and R6/2 SAHA and vehicle treated mice are shown. HDAC1, HDAC3, HDAC5, HDAC7 and HDAC9 protein levels do not change upon SAHA treatment of WT or R6/2 mice in the cortex (A) nor in the brain stem or hippocampus (data not shown). In all cases, HDAC protein levels were quantified using densitometry and normalized to alpha-tubulin. Error bars represent S.E.M. (*n* = 4).

**Figure 5:**
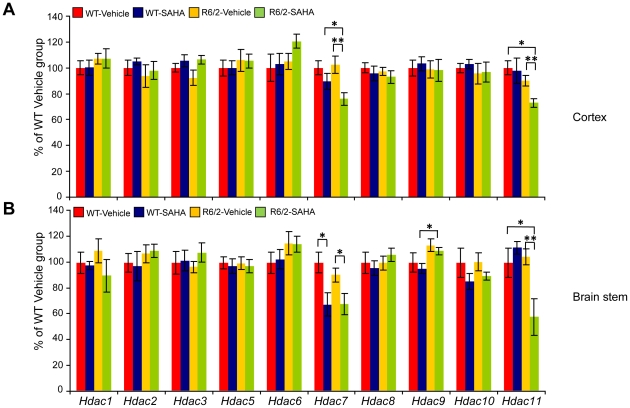
Of the 11 HDACs, SAHA specifically down-regulates *Hdac7* and *Hdac11* at the mRNA in WT and R6/2 mice – 2^nd^ trial. *Hdac7* and *Hdac11* transcript levels were reduced in the cortex (A) and brain stem (B) of R6/2 mice treated with SAHA. *Hdac* expression levels are shown as relative expression ratios to the geometric mean of three housekeeping genes in 9 week-old mouse WT vehicle (red bars), WT SAHA (blue bars), R6/2 vehicle (yellow bars) and R6/2 SAHA (green bars) mice. Error bars are S.E.M (n = 6). * p<0.05, ** p<0.01, *** p<0.001.

Next, we investigated whether chronic SAHA treatment can affect the protein levels of other HDACs, especially the members of the class IIa group. First, we screened a number of commercially available antibodies raised against HDAC enzymes and found that only antibodies for HDACs: 1, 2 and 3 (class I) and HDACs: 5, 7 and 9 (class IIa) were suitable for immunodetection in mouse tissues. We found that SAHA had no effect on the protein levels of HDACs: 1, 3, 5, 7 and 9 in the cortex (Fig. 4A), brain stem (data not shown) or hippocampus (data not shown) of WT and R6/2 mice in comparison to their vehicle groups. Suprisingly, we found that like HDAC4, SAHA decreased HDAC2 protein levels in cortex (Fig. 4A) and brain stem (Fig. 4B) but not hippocampus of the WT and R6/2 mice (data not shown). To investigate the overall relationship between HDAC mRNA and protein levels, we profiled the transcript levels of all 10 remaining HDACs by qPCR in the cortex and brain stem. As had been found previously [30],[31], SAHA significantly decreases *Hdac7* transcript levels in both brain regions (Fig. 5A, 5B). However, the attenuation of *Hdac7* mRNA levels was more pronounced in the brain stem for both WT and R6/2 mice than in the cortex (Fig. 5), likely due to the heterogeneity of cortical tissues. In addition, we discovered that *Hdac11* mRNA levels are decreased upon SAHA treatment in the cortex and brain stem of R6/2 mice but not WT mice (Fig. 5). In conclusion, SAHA treatment resulted in a reduction in HDAC2 and HDAC4 at the protein but not RNA level in some brain regions and that *Hdac*7 and *Hdac*11 were modulated at the RNA level.

## Discussion

Suberoylanilide hydroxamic acid, known as SAHA or Vorinostat, was the first HDAC inhibitor to be approved for advanced cutaneus T-cell lymphoma cancer therapy [32]. In addition, we have shown SAHA to be a potent modulator of HD-related phenotypes in the R6/2 mouse [20]. SAHA is predominantly an inhibitor of class I HDACs [23]. However, it can also bind to class IIa HDACs [24],[25] and has been shown to degrade class IIa HDACs at the protein level *in vitro* [26]. Thus, in this study we asked whether chronic treatment of SAHA might result in the degradation of class IIa HDACs in both wild type and R6/2 mice *in vivo*.

We had previously found that SAHA could be solubilised by complexing it with hydroxypropyl-β-cyclodextrins [20], a process that required boiling the reagents, and could conceivably affect its stability. Therefore, we assessed the stability and purity of SAHA in this formulation when incubated at room temperature for a period of up to seven days. We were reassured to find that the concentration of SAHA did not change during this period and that its purity remained at >99% (Fig. S1A). Similarly, the brain pharmacokinetic profile of SAHA has not previously been published. As expected we found that SAHA was brain penetrant, but was cleared rapidly from both plasma and brain and that the brain∶plasma ratio was <0.12 at most time points (Fig. S1B).

SAHA was administered to wild type and R6/2 mice at a concentration of 0.67 mg/ml in drinking water over a period of 4 weeks. We performed western blotting on lysates from the cortex, brain stem and hippocampus to determine the levels of the class I enzymes: HDACs 1–3 and all of the class IIa HDACs. We found that HDAC2 and HDAC4 levels decreased upon SAHA treatment to approximately 30% in the cortex and brain stem but not in the hippocampus of both WT and R6/2 mice. This discrepancy between brain regions might be explained by brain-region dependent exposure to SAHA, or at least in the case of HDAC4, due to its high hippocampal expression level [33]. Alternatively, as HDAC inhibitors have been shown to display different properties in various cell lines [34], the discrepancy could be due to variation in the cell-type composition of the brain regions. We did not detect changes in the protein level of the other class I (HDAC1 and 3) or class IIa (HDACs 5, 7 and 9) enzymes and were not able to assess protein levels of HDACs 6, 8, 10 and 11 due to the lack of available antibodies. To investigate whether the changes in HDAC2 and HDAC4 levels were a consequence of decreased gene expression, we prepared a set of qPCR assays to measure the transcript levels of all *Hdacs*. We found that the mRNA levels of *Hdac2* and *Hdac4* remained unchanged in SAHA treated tissues. Therefore, we might speculate that the mechanism through which SAHA leads to a reduction in HDAC4 is through RANBP2-mediated proteasome degradation as determined for cancer cell lines [26]. As previously described, we found that SAHA reduces *Hdac7* transcript levels [30],[31], although we have previously shown that the genetic reduction of *Hdac7* does not alleviate HD-phenotypes in the R6/2 mouse [31]. In addition, *Hdac11* mRNA levels were decreased in R6/2 but not in wild type cortex and brain stem upon SAHA treatment. Unfortunately, because of the lack of an HDAC11 antibody, we were unable to determine if this translated to the protein level. Interestingly, HDAC11 has been identified as a molecular target that controls immune activation versus immune tolerance through Interleukin 10 [35], an inhibitory cytokine that is up-regulated in HD *post mortem* brains [36].

We have recently developed the seprion-ligand ELISA to quantify the aggregate load in tissues samples from mouse models of HD [7]. This assay has good statistical power to detect small changes in aggregation that occur due to therapeutic interventions administered to R6/2 mice from five to nine weeks of age [37]. Therefore, we measured the aggregate load in the cortex, brain stem and hippocampus of R6/2 mice that had been treated with SAHA over this four week period. We found that there was a statistically significant reduction in aggregation in the cortex and brain stem, but not in the hippocampus, which correlates with the reduction in HDAC2 and HDAC4 levels. The hippocampal data are consistent with our previous finding that SAHA treatment did not inhibit polyQ aggregation in hippocampal slice cultures from R6/2 mice [20]. We also found that there was a partial restoration in the expression level of the *Bdnf* protein coding exon. Our *in vivo* finding, that SAHA increases expression of *Bdnf*, correlates with previous *in vitro* study in the neuron-glia cultures, indicating that HDAC inhibitors up-regulate *Bdnf* expression [38]. Moreover, we confirmed that the improvement in these molecular phenotypes had not arisen through a decrease in the expression of the R6/2 transgene.

HDAC inhibitors have been shown to consistently improve the phenotypes of HD mouse models [20],[39],[40] and are being developed as HD therapeutics. Our demonstration that the administration of SAHA to wild type and R6/2 mice decreases the levels of HDAC2 and HDAC4 at the protein and not RNA levels extends previous observations in cancer cell lines to the *in vivo* situation. In addition, a recent study has demonstrated that trichostatin A (TSA) and sodium butyrate also result in a reduction in HDAC4 when administered to embryoid bodies [41]. Consistent with the work described by Scognamiglio [26] they also show that this occurs through a proteasome-dependent mechanism [41] and arises as a general consequence of HDAC inhibitor action. Therefore, the beneficial effects of HDAC inhibitors in the context of HD might be more complex than previously anticipated and encompass decreased class I deacetylase activity, increased degradation of HDAC2 and/or HDAC4 and a reduction in the transcription of *Hdac11*.

## Materials and Methods

### Ethics statement

All experimental procedures performed on mice were approved by the King's College London Ethical Review Process Committee and carried out under the UK Home Office License 70/6545.

### SAHA formulation, purity and stability

For chronic administration via drinking water, SAHA was complexed at a 1∶5 molar ratio with hydroxypropyl-β-cyclodextrin 97% (Acros Organics, Cat. No.: 297561000, Lot no. A0272247) in deionised, filtered, irradiated water. SAHA was prepared at a concentration of 0.67 mg/ml in 5M hydroxypropyl-β-cyclodextrin and solubilisation was achieved by boiling for two minutes followed by cooling slowly to room temperature for approximately 2 hours. LC-MS/MS analysis was employed to ensure the purity and stability of the SAHA. For calibration, a 1.0 mg/ml [1.75 mg/1.75 ml] solution of solid SAHA [C14H20N2O3; MW 264.33] was prepared in 80/20 v/v acetonitrile/DMSO for LC-MS purity analysis after diluting with 100 µl of DMSO.

LC-MS full scan [150–850 m/z] analysis was performed using electrospray atmospheric pressure ionization source in positive ion mode (ESI+) with HP Agilent 1100 binary pump coupled to Waters ZQ single quadrupole mass spectrometer, Waters 2996 PDA and Waters 2420 ELS detectors. Analysis was performed on a Waters Atlantis dC18 column 2.1 mm×100 mm, 3 µm using a generic gradient with injection volume of 3 µl and flow rate of 0.6 ml/min with a total run time of 7 minutes. The mobile phase (solvent A) comprised of water +0.1% formic acid and (solvent B) acetonitrile +0.1% formic acid.

The UV purity of SAHA prepared from solid was >99%. SAHA was formulated at 0.67 mg/ml as above and stored at RT. Aliquots were removed on the day of formulation and after 2, 4 and 7 days and snap frozen. The concentration of SAHA remained unchanged over the seven day period and the UV purity remained >99% on day seven (Fig. S1A).

### Pharmacokinetic study

WT and R6/2 female mice were subcutaneously injected with a single dose of 200 mg/kg SAHA formulated with hydroxylpropyl-β-cyclodextrin as stated above and tissues were harvested at 0.25, 0.5, 1, 2, 4, 8, 16 and 24 hours post dosing. Mice were deeply anesthetised with Euthatal and blood was collected by cardiac puncture into K_2_EDTA collection tubes (Greiner Bio-One). Samples were centrifuged at 9600× *g* for 10 min, the supernatant was collected and frozen in liquid nitrogen. Brain tissues were immediately frozen in liquid nitrogen.

The concentrations of SAHA in the mouse plasma and brain were determined using an LC-MS/MS assay developed at BioFocus. The lower limit of quantification (LLOQ) was 19 nM for plasma and 3.8 nM for brain. Calibration standards for plasma (5.00–10000 ng eq./ml) and brain (1.00–10000 ng eq./g) were prepared by adding SAHA to control mouse plasma and brain samples respectively. Diclofenac was used as an internal standard in the LC-MS/MS assay, except for plasma, where a difference in the internal standard response was seen between the study and calibration samples. Therefore, the plasma data were generated without normalizing the analytic response for internal standard response. Calibration standards were prepared in duplicate for each concentration and at a minimum, 75% of all the calibration standards and at least one calibration standard per concentration met the accuracy and precision criteria of ±30%. There was no bias in the accuracy or precision of the calibration standards. The coefficient of variation (CV%) of the internal standard signal/area response for the entire run was within the criteria of ±30%, and there was no bias or trend in the internal standard signal/area response. Figure S1B shows the SAHA concentrations measured in the plasma and brain tissues at different time points.

### Mouse maintenance and breeding

Hemizygous R6/2 mice were bred by backcrossing R6/2 males to (CBA x C57BL/6) F1 females (B6CBAF1/OlaHsd, Harlan Olac, Bicester, UK). All animals had unlimited access to water and breeding chow (Special Diet Services, Witham, UK), and housing conditions and environmental enrichment were as previously described [42]. Mice were subject to a 12-h light/dark cycle. All experimental procedures were performed according to Home Office regulations.

### Genotyping and CAG repeat sizing

Genomic DNA was isolated from an ear-punch. R6/2 mice were genotyped by PCR as previously described and the CAG repeat was measured by sequencing with an ABI3730 automated sequencer as previously described [7].

### Chronic SAHA dosing regime

In the case of both trials, female WT and R6/2 mice were randomized from litters born over a period of 1 day. The number of animals per treatment group and CAG repeat size are summarised in Table S1. Mice were weaned into their treatment groups at four weeks of age. Both trials were initiated at 5 weeks of age and SAHA (0.67 mg/ml) or vehicle was administrated in drinking water for 4 consecutive weeks (bottles with SAHA or vehicle were changed on the weekly basis). Trials were terminated at 9 weeks of age and all tissues were dissected, snap frozen in liquid nitrogen and stored at −80°C until further analysis.

### RNA extraction and Taqman real-time PCR expression analysis

Total RNA from cortex and brain stem was extracted with the mini-RNA kit according to manufacturer instructions (Qiagen). The reverse transcription reaction (RT) was performed using MMLV superscript reverse transcriptase (Invitrogen) and random hexamers (Operon) as described elsewhere [28]. The final RT reaction was diluted 10-fold in nuclease free water (Sigma) for further Taqman-qPCR reactions. All Taqman-qPCR reactions were performed as described previously [28], using the Chromo4 Real-Time PCR Detector (BioRad). Estimation of mRNA copy number was determined in triplicate for each RNA sample by comparison to the geometric mean of three endogenous housekeeping genes (Primer Design) as described [28]. Primer and probe sequences for HDACs 1–11 are presented in Table S2 and were purchased from Operon. Primer and probe sequences for *Bdnf* and *mt-exon 1 HTT* were described previously [28].

### Protein extraction from snap frozen tissues for Immunoblotting and ELISA

Cortical, hippocampal and brain stem tissues were stored at −80°C. All protein lysates from tissues examined in this study were prepared as 2.5% lysates (w/v) by homogenizing tissue with ceramic beads (MP Biomedicals, lysing matrix D) in RIPA buffer (1% NP-40, 0.5% deoxycholate, 0.1% SDS, 50 mM Tris-HCl pH 8.0, 150 mM NaCl, 1 mM β-mercaptoethanol, 100 µM PMSF, 1 mM DTT) supplemented with protease inhibitor cocktail (Roche). Homogenization was achieved by 2 runs for 30 seconds at 6.5 meters/second using a ribolyser (Fast Prep-24, MP Biomedicals). In between these cycles, the samples were placed on ice for 5 minutes. Next, samples were snap frozen on dry ice and thawed. At this stage, samples were used for the seprion-ligand ELISA to capture huntingtin aggregates. For immunoblotting, samples were sonicated with a Vibracell sonicator (Sonics & Material Incorporated) at 40 Hz, with 10 pulses (1 second each), and placed on a spinning wheel at 4°C for 15 min. Purification was achieved by spinning the samples at 16,200× *g* for 15 min at 4°C. Protein concentration was measured using the Pierce BCA assay kit (Thermo Scientific).

### Antibodies and western blotting

20 µg protein lysate was fractionated on a 10% SDS-PAGE gel and transferred to a Protran nitrocellulose membrane (Whatman) by submerged transfer apparatus (Bio-Rad) in transfer buffer (25 mM Tris-HCl, 192 mM glycine, 20%, v/v, methanol). 150 ug protein lysates was used for HDAC7 detection. Membranes were blocked for at least 30 minutes at room temperature in 0.1% PBS-Tween20 (PBST) containing 5% non-fat dried milk. Next, membranes were incubated with primary antibodies in 0.1% PBST containing 1% non-fat dried milk with gentle agitation over night at 4°C. The following primary antibodies were used: HDAC1 (1∶1000) from Sigma (H3284), HDAC2 (1∶1000) from Sigma (H3159), HDAC3 (1∶1000) from Millipore (clone Y415), HDAC4 (1∶1000) from Santa Cruz (SC11418), HDAC4 (1∶500) Sigma (H9536), HDAC5 (1∶1000) from Abcam (ab1439), HDAC7 (1∶250) as described [31], HDAC9 (1∶1000) from Biovision (3609-100). α-tubulin (1∶40,000) from Sigma was used as a loading control. For chemiluminescent detection, blots were washed three times in 0.1% PBST, then probed with HRP-linked secondary antibodies (HRP conjugated anti-rabbit (1∶20,000, Perbio) or anti-mouse (1∶5000, Daco) in 0.1% PBST for 1 hour at RT followed by washing in 0.1% PBST. To detect signal by chemiluminescense, the GE Healthcare Enhanced detection kit and Amersham Hyperfilms (both from GE Healthcare) were used according to the manufacturer's instructions. The signals were quantified using a GS-800 calibrated densitometer (Bio-Rad).

### Seprion ligand ELISA

Aggregates were captured in seprion-ligand coated plates (Microsens) and detected with the S830 sheep polyclonal antibody as described [7].

### Statistical analysis

All data were analysed with Microsoft Office Excel and Student's *t*-test (two tailed).

## Supporting Information

Figure S1 **Pharmacokinetic study and SAHA stability.** SAHA shows long-term stability when complexed with hydroxypropyl-β-cyclodextrin in water (A). The pharmacokinetic study revealed SAHA to be a brain penetrant compound with fast clearance characteristics (B).
(TIF)

Table S1 **Number of animals per treatment group and CAG repeat size.**
(DOC)

Table S2 **Primers and probes sequence for** ***Hdacs*** **1–11.**
(DOC)
