# Supplementary material for: SAHA Decreases HDAC 2 and 4 Levels In Vivo and Improves Molecular Phenotypes in the R6/2 Mouse Model of Huntington's Disease
Source: PLoS One. 2011 Nov 28;6(11):e27746. doi: 10.1371/journal.pone.0027746 (PMC3225376; doi:10.1371/journal.pone.0027746)
Supplement: Table S1 — Number of animals per treatment group and CAG repeat size. (DOC) [file pone.0027746.s002.doc]

| **Dose** | **WT** | **R6/2** | **CAG repeat**  **(mean ± s.d.)** | **Trial** |
| --- | --- | --- | --- | --- |
| vehicle | 7 | 9 | 194 ± 2.66 | 1 |
| 0.67mg/ml SAHA | 5 | 5 | 197 ± 4.16 |
| vehicle | 8 | 8 | 211 ± 12.9 | 2 |
| 0.67mg/ml SAHA | 6 | 8 | 207 ± 3.3 |
