# Supplementary material for: SAHA Decreases HDAC 2 and 4 Levels In Vivo and Improves Molecular Phenotypes in the R6/2 Mouse Model of Huntington's Disease
Source: PLoS One. 2011 Nov 28;6(11):e27746. doi: 10.1371/journal.pone.0027746 (PMC3225376; doi:10.1371/journal.pone.0027746)
Supplement: Table S2 — Primers and probes sequence for Hdacs 1–11. (DOC) [file pone.0027746.s003.doc]

| **Gene** | **Forward Primer** | **Probe (5’FAM, 3’TAMRA)** | **Reverse Primer** |
| --- | --- | --- | --- |
| ***Hdac1*** | 5’>TCTGAATACAGCAAGCAGATGCA>3’ | 5’>AGATTCAATGTTGGTGAGGACTGTCCGG>3’ | 5’>ACAGAACTCAAACAAGCCATCAAAC>3’ |
| ***Hdac2*** | 5’>AGAAGATTGTCCGGTGTTTGATG>3’ | 5’>TTGAGTTTTGTCAGCTCTCCACGGGTG>3’ | 5’>CACAGCCCCAGCAACTGAA>3’ |
| ***Hdac3*** | 5’>TCAGCCCCACCAATATGCA>3’ | 5’>CCTTAATGCCTTCAACGTGGGT>3’ | 5’>GAACTCGAAAAGTCCTGGAAACA>3’ |
| ***Hdac4*** | 5’>CTGGCATCCCTGTGTCATTTG>3’ | 5’>CTGCCACCTTCCCCATGTCAGTCC>3’ | 5’>ACACAAGACCTGTGGTGAACCTT>3’ |
| ***Hdac5*** | 5’>GCAACAAGGAGAAGAGCAAAGAG>3’ | 5’>TGCCATCGCCAGCAC>3’ | 5’>TCCTGGAGCCTCAGCTTTACC>3’ |
| ***Hdac6*** | 5’>CTGCATGGCATCGCTGGTA>3’ | 5’>TTCCCATCGCCTATGAGTTTAACCCAGAAC>3’ | 5’>GCATCAAAGCCAGTGAGATC>3’ |
| ***Hdac7*** | 5’>CCCACCTGTCAGACCCAAGT>3’ | 5’>CTCAACAGCTCAGAGACA>3’ | 5’>AGTCATAGACCAGCCCTGTAGCA>3’ |
| ***Hdac8*** | 5’>GGCCCATCCATCCCTGTAG>3’ | 5’>TGGACGAGGGACCAGG>3’ | 5’>TTTAGATCGCCGGAGACAGTTT>3’ |
| ***Hdac9*** | 5’>TGGCAGAATCCTCGGTCAGT>3’ | 5’>TCTCCAGGGTCAGGTCCCAGTTCACC>3’ | 5’>CCCAGCAGGGCCATTGT>3’ |
| ***Hdac10*** | 5’>CCGCTATGAGCATGGAAGCT>3’ | 5’>CTGGCCGTTTCTC>3’ | 5’>CAACTGCATCTGCATCAGACTCT>3’ |
| ***Hdac11*** | 5’>TGGGCATGAGCGAGACTTC>3’ | 5’>TGGGTGACAAGCGAG>3’ | 5’>GCGGTTGTAAACATCCATGATG>3’ |
